# *Haemophilus parasuis* cytolethal distending toxin induces cell cycle arrest and p53-dependent apoptosis

**DOI:** 10.1371/journal.pone.0177199

**Published:** 2017-05-18

**Authors:** Gang Li, Hui Niu, Yanhe Zhang, Yanling Li, Fang Xie, Paul R. Langford, Siguo Liu, Chunlai Wang

**Affiliations:** 1 State Key Laboratory of Veterinary Biotechnology, Division of Bacterial Diseases, Harbin Veterinary Research Institute, Chinese Academy of Agricultural Sciences, Harbin, China; 2 Section of Paediatrics, Department of Medicine, Imperial College London, St. Mary’s Campus, London, United Kingdom; Cornell University, UNITED STATES

## Abstract

*Haemophilus parasuis* is the causative agent of Glasser’s disease in pigs. Cytolethal distending toxin (CDT) is an important virulence factor of *H*. *parasuis*. It is composed of three subunits: CdtA, CdtB and CdtC and all were successfully expressed in soluble form in *Escherichia coli* when the signal peptides were removed. Purified CdtB had DNase activity, i.e. caused DNA double strand damage, in vitro and in vivo prior to cell arrest and apoptosis. Flow cytometry analysis showed CdtB alone could induce cell cycle arrest and apoptosis in PK-15 porcine kidney and pulmonary alveolar macrophage (PAM) cells, which could be enhanced by CdtA or/and CdtC. CDT holotoxin could lead to significant cell distension, G_2_ arrest and apoptotic death in PK-15 and PAM cells. The apoptosis induced by CDT holotoxin was significantly inhibited by pifithrin-α, which indicates that it is p53-dependent. The results suggest that *H*. *parasuis* CDT holotoxin is a major virulence factor.

## Introduction

*Haemophilus parasuis* is a small, Gram-negative nicotinamide adenine dinucleotide-dependent bacterium which is a member of the family *Pasteurellaceae*. The bacterium colonises the upper respiratory tract of pigs but is also the aetological agent of Glasser’s disease, which presents clinically as fibrinous polyserositis, polyarthritis and/or meningitis. To date, 15 *H*. *parasuis* serovars with different virulence potential have been described [1]. Prevalent serovars exhibit diversity in different countries and regions [1–4]. *H*. *parasuis* infection causes significant mortality and morbidity and is responsible for enormous economic losses in the swine industry [5]. However, the molecular mechanisms by which the bacterium interacts with the host and cause pathogenicity are largely unknown. The subject of this study is the cytolethal distending toxin of *H*. *parasuis* (HparCDT) [6], a virulence factor that has been reported to facilitate attachment to host cells and evade the immune system.

The cytolethal distending toxins (CDTs) consists of a family of bacterial protein exotoxins, associated with the pathogenesis of a diverse group of bacteria capable of causing disease. A variety of Gram-negative pathogenic bacteria produce CDTs, e.g. *Campylobacter jejuni*, *Haemophilus ducreyi*, *Aggregatibacter actinomycetemcomitans*, *Helicobacter hepaticus*, *Escherichia coli*, *Shigella dysenteriae* and *H*. *parasuis* [7–12]. All CDT holotoxins are tripartite complexes comprising CdtA, CdtB, and CdtC subunits [13], CdtA and CdtC subunits are essential proteins for mediating toxin binding to the plasma membrane of target cells, allowing the internalization of the main active subunit CdtB which is functionally homologous to mammalian deoxyribonuclease I [14]. CdtB is thus important for deleterious effects on host cells.

CDT has been described as the first bacterial genotoxin whose main action is activating the DNA damage responses, inducing cell cycle arrest and apoptosis of host cells [15]. *H*. *parasuis* has two copies of CDTs that possess the same toxin activity in vitro [16]. Recent research showed that HparCDT enhanced *H*. *parasuis* adherence to and invasion of the host cells [17]. However, the mechanism by which HparCDT causes cell cycle arrest and apoptosis of host cells has not been described. In this study, we show that the p53 signaling pathway plays an important role in cell cycle arrest and apoptosis caused by HparCDT.

## Materials and methods

### Cell lines, bacterial strains

Porcine alveolar macrophage (PAM) and kidney epithelial (PK-15) cell lines were obtained from ATCC, and both were cultured with Dulbecco's Modified Eagle Medium (DMEM) (Hyclone) containing 10% heat inactivated fetal bovine serum (FBS) (Gibco) and maintained at 37°C in 5% CO_2_. The *H*. *parasuis* serovar 5 reference strain Nagasaki was cultured in tryptic soy broth (TSB) (Difco) or on tryptic soy agar (TSA) supplemented with 10 μg/ml NAD and 5% equine sera (Gibco), and was incubated at 37° C in a 5% CO_2_ incubator [18].

### Expression and mutagenesis of *Cdt* genes and purification of recombinant proteins

The genomic DNA of *H*. *parasuis* strain Nagasaki was extracted from bacterial suspension in sterile phosphate-buffered saline with a bacterial genomic DNA extract kit (Tiangen, China) according to the manufacturer’s instructions. The *cdtA*, *cdtB*, and *cdtC* genes without the 5′-terminal signal peptide sequences were obtained by PCR with the genomic DNA of *H*. *parasuis* strain Nagasaki as the template. The PCR primers for the *cdt* genes are shown in Table 1. The restriction enzyme sites were marked by underscore. PCR products were digested with EcoRI and XhoI and ligated to EcoRI and XhoI digested pET-22b(+) vector resulting inthe recombinant plasmids, pET-22b-*cdtA*, pET-22b-*cdtB*, and pET-22b-*cdtC*.

**Table 1:** Primers for cloning *cdt* genes.

Gene	Primer	Sequence (5'-3')
*cdtA*	sense	CGgaattcTGGTTGCAGTTGTAGTTGTG
	anti-sense	ATctcgagTAATGGATTAGCACTAAGTAATG
*cdtB*	sense	CGgaattcTAATTTGGAAAACTATACGG
	anti-sense	ATctcgagACGTTTTTTTACAAAGCTG
*cdtC*	sense	CGgaattcTTGCCAGTTTTCCCTTGCAG
	anti-sense	ATctcgagTAATAACCTACTAGGCCC

*E*. *coli* BL21(DE3) *plysS* (Biomed, China) harboring the pET-22b-*cdtABC* plasmids were cultured in 0.5 l of LB medium containing kanamycin (50 μg/ml) until the OD_600_ reached 0.6. Isopropyl-β-D-thiogalactopyranoside (IPTG) was added to a final concentration of 1 mM, and the cells were cultivated further at 30°C overnight.

Cells were harvested by centrifugation at 5,000×*g* for 15 min at 4°C and lysed by sonication in Tris-HCl buffer (pH 8.0) supplemented with 0.1 mM phenylmethanesulfonyl fluoride (PMSF) immersed in ice water. The clear lysate was centrifugated at 12,000×*g* for 20 min at 4°C, and recombinant proteins purified from the supernatant with Ni-NTA agarose (QIAGEN). The predicted molecular mass of the purified recombinant proteins was confirmed by sodium dodecyl sulfate-polyacrylamide gel electrophoresis (SDS-PAGE) and Western blotting using a mouse anti-His tag monoclonal antibody (Tiangen, China) as the primary antibody, horseradish peroxidase (HRP)-conjugated goat anti-mouse IgG (1:5000) (Sigma, USA) as the secondary antibody and detection carried out by using the diamino benzidine detection reagent (Tiangen, China).

Based on sequence homology analysis and previous studies[14], the active site of CdtB was predicted to be histidine 161. Therefore, glutamine substitution mutagenesis was done using the QuikChange® Site-Directed Mutagenesis Kit (Stratagene, USA). The recombinant plasmid, pET-22b-*cdtB*, was used as the template. The primers used for mutagenesis are shown in Table 2. The mutagenesis PCR products were transformed into *E*. *coli* strain DH5α (Tiangen, China) directly after DpnI digestion. The plasmid harboring the histidine to glutamine codon mutation in *cdtB* was confirmed by sequencing and transformed into *E*. *coli* BL21(DE3) *plysS* (Biomed, China). Purified His_6_-tagged mutant CdtB^H161Q^ was expressed and purified as described above for the wild-type protein.

**Table 2:** Primers used for site-directed mutagenesis in this study.

Amino acid substitution	Base change (codon)	Sense (5′→3′)	Anti-sense (5′→3′)
H161Q	CAT→CAG	CTTTAGTATTCAGGCTCTTTCATCTGGAGGAG	CTCCTCCAGATGAAAGAGCCTGAATACTAAAG

### DNase activity assay

CdtB or CdtB^H161Q^ were analyzed for DNAase activity by adding 2 μg of recombinant protein in 20 μl of MgCl_2_ buffer (25 mM HEPES, pH 7.0, 10 mM MgCl_2_, and 5 mM CaCl_2_) to 1 μg of pET-22b (supercoiled) or SalI linearized plasmid and incubating for 37 ^o^C for 1 h[19]. DNase I (1 mg/ml, Sigma) was used as positive control and MgCl_2_ buffer was used as negative control. Ten μl of each sample was loaded onto a 1% agarose gel, electrophorised and stained by Ethidium bromide.

### Laser confocal assay

PK-15 cells (1–5×10^6^) were incubated with 500 ng/ml of recombinant proteins for 12 h in 12-well tissue culture plates (Nest, China), washed 3 times with PBS, cells fixed with 4% paraformaldehyde in PBS for 20 min and permeabilized with 0.2% Triton X-100 for 15 min. After blocking with 3% FBS in PBS for 1 h, the cells were incubated with rabbit anti-γH2A.X (phospho S139) (Abcam) overnight at 4°C. washed 5 times with PBST, and incubated with anti-rabbit IgG (H + L)-FITC antibody produced in goat (Sigma) at 37°C for 1 h. Nuclei were stained with 4, 6-diamidino-2-phenylindole (DAPI) (Beyotime) for 15 min and the γ-H2A.X foci examined in a Leica SP2 Confocal system (Leica Microsystems, Germany).

### γ-H2A.X flow cytometry

PK-15 cells seeded into 12-well tissue culture plates treating with 500 ng/ml of recombinant proteins for 24 h were harvested, and approximately 1–5×10^6^ cells were fixed with 75% cold ethanol on ice for 2 h, and permeabilized with 0.2% Triton X-100 for 15 min. After blocking with 3% FBS in PBS for 1 h, the cells were incubated with rabbit anti-γH2A.X (phospho S139) overnight at 4°C. Following washing 3 times with cold PBS, the cells were incubated with goat anti-rabbit IgG (H + L)-FITC antibody for 1 h at 4°C in the dark, washed a further 3 times and resuspended in 200 μl cold PBS. Then cell suspensions were immediately stored at 4°C in the dark and analyzed in the BD_FACSAria_III flow cytometer.

### Cell cycle analysis

PAM or PK-15 cells were treated with 500 ng/ml of recombinant proteins for 24 h, trypsinized, centrifuged, and washed once with PBS. The cell pellet was resuspended and fixed with cold ethanol for 2 h on ice. After removal of RNA with DNase-free RNase (0.02mg/ml), the cells were subsequently centrifuged and resuspended in 1 ml of propidium iodide (PI) solution for 1 h at 4°C. Flow cytometry analysis was performed on BD_FACSAria_III flow cytometer.

### Cell apoptosis analysis

Apoptotic cells were quantified by flow cytometry using an Annexin-V-FITC/PI Apoptosis Detection Kit (BD Biosciences) following the manufacturer’s instructions. Briefly, cells were treated with 500 ng/ml of recombinant proteins for 36 h, collected and washed twice with cold PBS followed by resuspensionwith 500 μl of Annexin-V binding buffer containing 5 μl of fluorescein isothiocyanate (FITC)-labeled Annexin-V, transfer into round-bottom tubes and incubated for 15 min in the dark. Finally, 5 μl of PI were added and the percentage of apoptotic cells measured by flow cytometry.

### Real-time qPCR

Cells were treated with CDT holotoxin and/or pifithrin-α (PFT-α) for 36 h, and total RNA isolated using TRIzol (Invitrogen) following the manufacturer’s instruction. For cDNA preparation, 2 μg of total RNA was added to 20 μl of reaction mixture containing 200 U (1μl) of Reverse Transcriptase XL (AMV) (Takara), 4 μl of 5 × Reverse Transcriptase XL Buffer, 2.5 μM Oligo dT-Adaptor Primer, 1 mM dNTPs and 20 U of recombinant RNase inhibitor. The reaction conditions were 25°C for 10 min, 42°C for 60 min and 75°C for 15 min. Subsequently real-time PCR was performed using UltraSYBR Mixture (CW biotech). The gene specific primers for real-time PCR used in this study are shown in Table 3. Reaction mixture (25 μl) contained 1× UltraSYBR Mixture, sense and anti-sense primers (0.4 mM) and target cDNA (4 ng). The cycling conditions were 95°C for 10 min, followed by 40 cycles of 95°C for 15 s, 60°C for 20 s and 72°C for 25 s. The *gapdh* gene was used as an endogenous control.

**Table 3:** Primers for real-time PCR.

Gene	Primer	Sequence (5'-3')
*bcl-2*	sense	GCCCCTGGTGGACAACATCGC
	anti-sense	CCACCAGGGCCAGACTGAGC
*bcl-xl*	sense	GGCCACTTACCTGAATGACCACC
	anti-sense	TCATGCCCGTCAGGAACCAT
*casp-3*	sense	CCGGAATGGCATGTCGAT
	anti-sense	TGAAGGTCTCCCTGAGATTTTGC
*p21*	sense	CGACCAGGGATGCACATCAGA
	anti-sense	GCACACGTTCCCAGGCGAAG
*gapdh*	sense	ACATGGCCTCCAAGGAGTAAGA
	anti-sense	GATCGAGTTGGGGCTGTGACT

### Western blot analysis

PK-15 cells were cultured in 6-well plates and were exposed to 500 ng/ml recombinant protein for 24 h (for detection of γ-H2A.X) or 36 h (for detection of cleaved caspase-3). The cells were lysed in lysis buffer (Beyotime) with protease inhibitor cocktail (Roche) for 30 min on ice and centrifuged at 14000×*g* for 10 min. Protein concentration was measured with the BCA protein assay kit (CWbiotech). Samples were resolved by SDS-PAGE and transferred to Nitrocellulose membranes (PALL). The membranes were blocked with 5% nonfat milk in PBS buffer containing 0.05% Tween-20, and incubated overnight at 4°C with primary antibodies against anti-γ H2A.X (phosphor S139), cleaved caspase-3 (CST) or GAPDH (CWbiotech). IRDye® 680RD goat anti-mouse IgG (H + L) or IRDye^®^ 800CW donkey anti-Rabbit IgG (H+L) (LI-COR bioscience) were used as secondary antibodies as appropriate according to the manufacturer’s instructions. Western blots were imaged using an Odyssey CLx imager (LI-COR bioscience). Quantification was performed on single channels with the analysis software provided.

### Statistical analysis

Statistical analyses were conducted using SPSS 13.0 software. Student’s *t*-test and One-way analysis of variance (ANOVA) was used to compare the percentage of G2 phase cells, apoptotic cells or relative mRNA change folds. A *P* value of <0.05 was considered significantly different.

## Results

### Expression and purification of recombinant Cdta, Cdtb, and Cdtc fusion proteins

SIGNAL-BLAST [20] analysis indicated that the first 19, 20 and 19 aa of the N-termini of CdtA, CdtB and CdTC, respectively are signal peptides. Both Predsi (http://www.predisi.de/home.html) and Signal-3L [21] (http://www.csbio.sjtu.edu.cn/bioinf/Signal-3L/) analysis indicate that the first N-terminal 19 aa and of CdtA and CdtC, and 21 aa of CdtB are signal peptides (S1 Fig).

**Fig 1:**
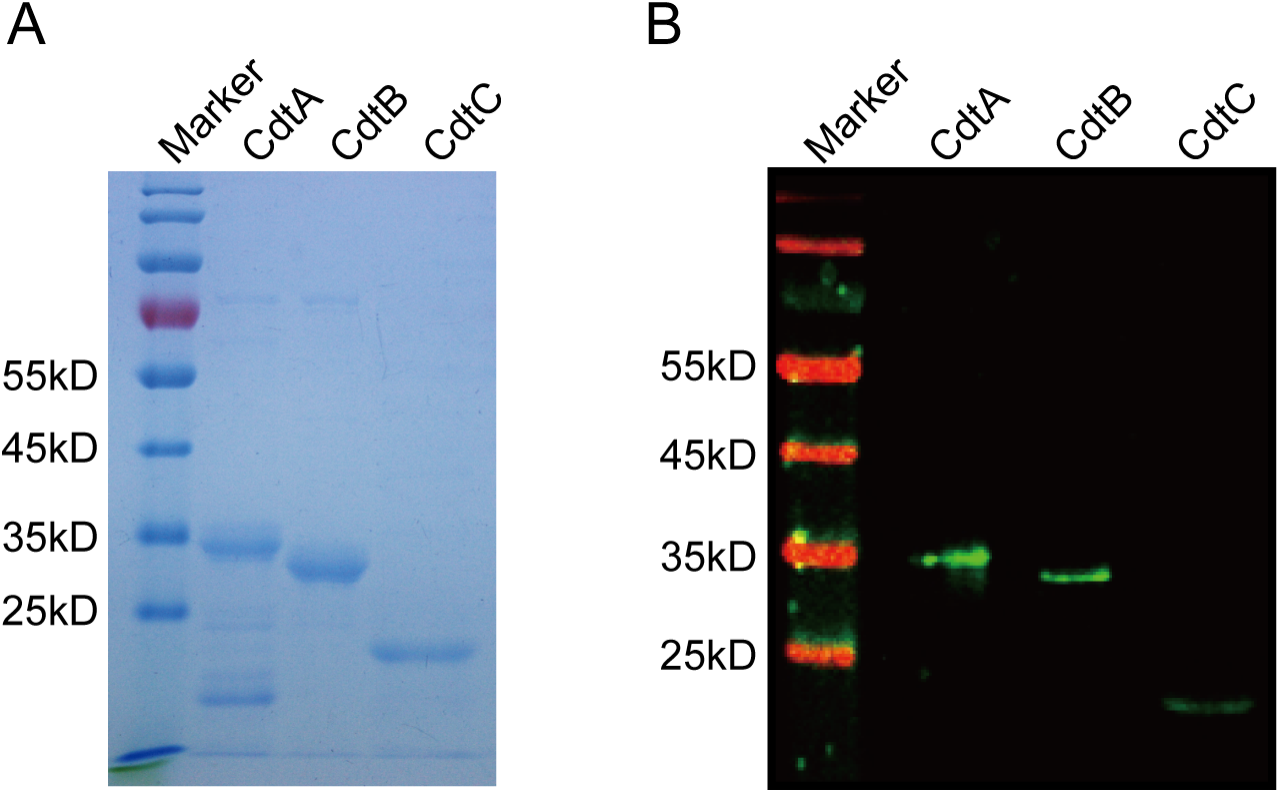
Expression and purification of CDT. (A) Sodium dodecyl sulfate-polyacrylamide gel electrophoresis (SDS-PAGE) analysis of purified CDT subunits. (B) Western blot analysis of purified CDT subunits using anti-His antibody.

Based on the signal peptide prediction results, three pair primers (Table 1) were designed to clone the *cdtA*, *cdtB*, and *cdtC* genes of *H*. *parasuis* strain Nagasaki each additionally encoding a His_6-_tag in the C-terminus. The expected molecular masses of purified recombinant His_6_-tagged fusion protein subunits without signal peptides were approximately 34 kDa for CdtA, 32 kDa for CdtB, and 20 kDa for CdtC, and these were confirmed by SDS-PAGE (Fig 1A). The identity of each protein was confirmed by Western blotting with anti-His_6_ antibody (Tiangen, China) (Fig 1B). These results showed that the three CDT subunits were of the expected molecular mass, and each expressed protein preparation was considered of sufficient purity for further experiments.

### CdtB has DNase activity in vitro and in vivo

**Fig 2:**
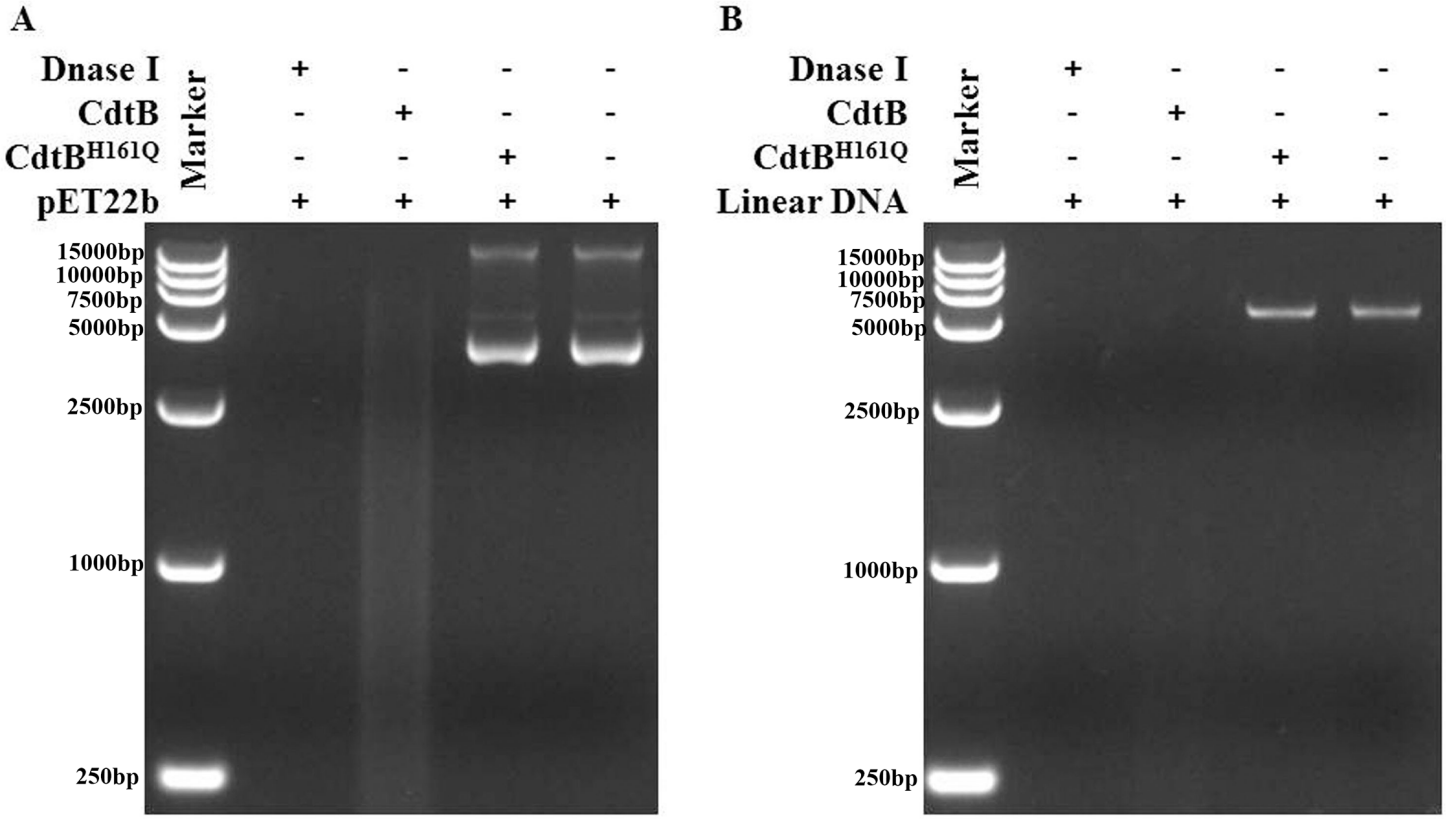
The DNase activity of CdtB. (A) Circular plasmids were incubated with CdtB for 1 h at 37°C and were electrophoresed on an agarose gel and stained with ethidium bromide (EB). (B) Linear plasmids were incubated with CdtB for 1 h at 37°C and analyzed by electrophoresis and staining.

Sequence homology analysis revealed that CdtB belongs to the Exonuclease-Endonuclease-Phosphatase (EEP) domain superfamily and predicted to have DNase activity. To identify whether CdtB has DNase activity, supercoiled circular plasmid (pET-22b) and linear plasmid (digested by SalI) was incubated with purified CdtB in MgCl_2_ buffer at 37°C for 1 h, and the products analyzed by electrophoresis. The result showed that both supercoiled (Fig 2A) and linear (Fig 2B) plasmid was digested by CdtB. In contrast, the mutant CdtB^H161Q^ (S2 Fig) did not digest either the supercoiled nor linearized plasmid. These data show that CdtB has DNase activity in vitro.

**Fig 3:**
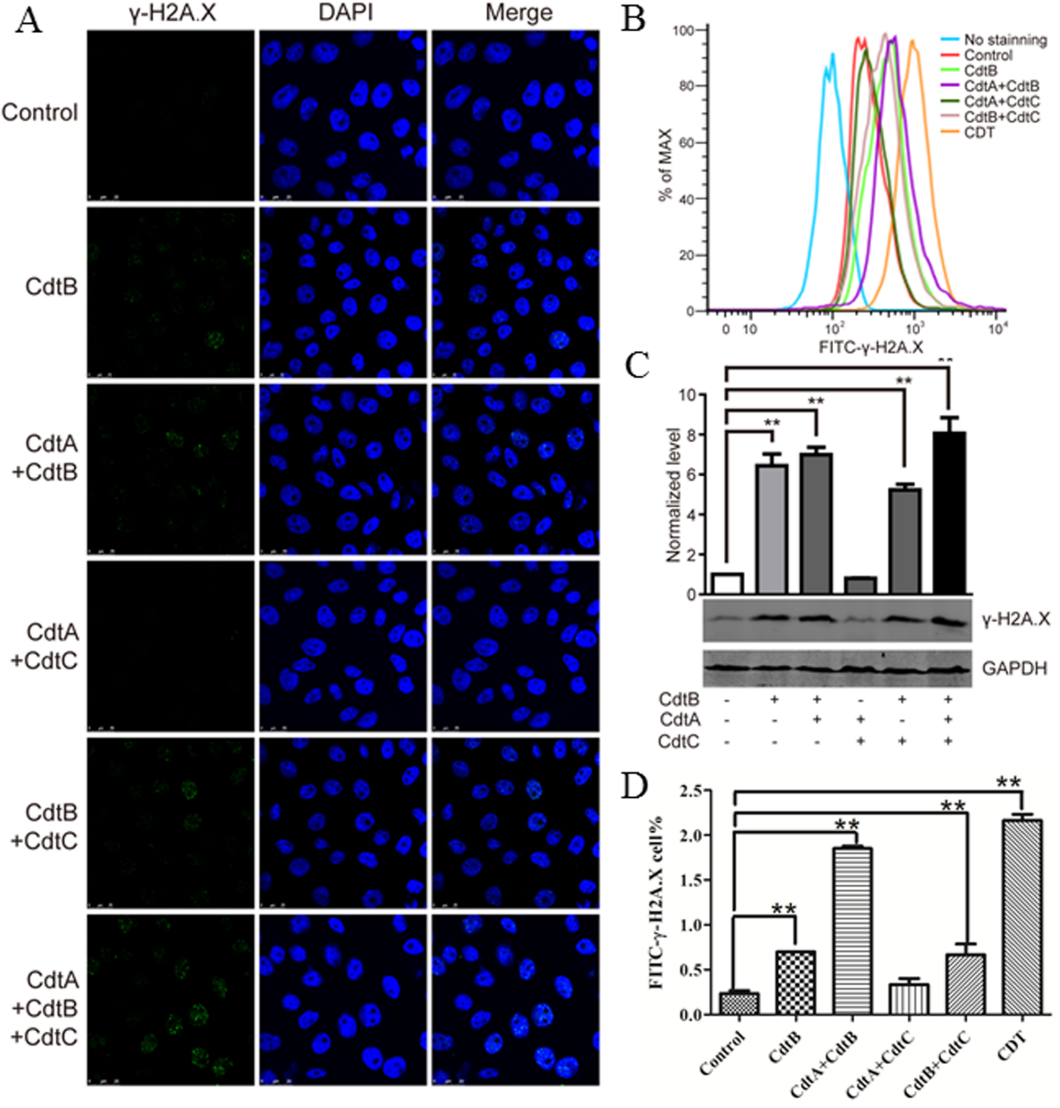
CdtB activated gamma-H2A.X. (A) PK-15 cells treated with or without CdtB supplemented with CdtA or CdtC for 24 h as shown, fixed and immuno-stained with γ-H2A.X antibody and DAPI, and then γ-H2A.X foci (green) observed under a confocal microscope. Scale bar corresponds to 200 μm. (B) Flow cytometry analysis of γ-H2A.X in PK-15 cells treated with or without CdtB supplemented with CdtA or CdtC for 24 h as shown, right shift of median fluorescence indicate a net increase of γ-H2A.X. (C) Quantitative Western blot analysis of γ-H2A.X in PK-15 cells treated with or without CdtB supplemented with CdtA or CdtC for 24 h as shown, graphs show normalized level of γ-H2A.X, cells treated without CDT were set as 1. (D) Quantitative analysis of γ-H2A.X in PK-15 cells treated with or without CdtB supplemented with CdtA or CdtC for 24 h by Flow cytometry.

**Fig 4:**
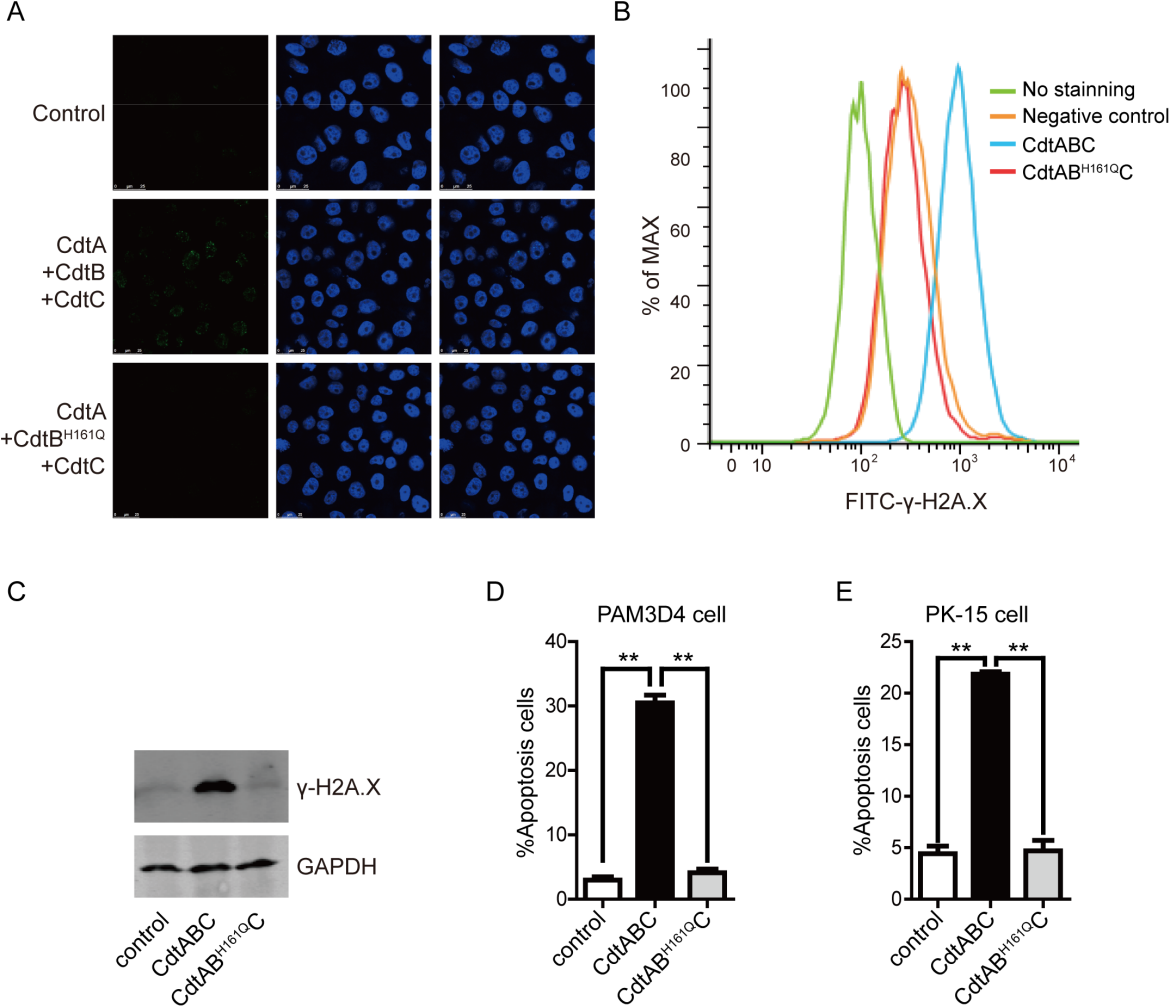
CdtB^H161Q^ has lost its ability to activate gamma-H2A.X. (A) PK-15 cells treated with CdtABC or CdtAB^H161Q^C for 24 h as shown were fixed, immuno-stained with γ-H2A.X antibody and DAPI, and γ-H2A.X foci (green) observed under a confocal microscope. Scale bar corresponds to 200 μm. (B) Flow cytometry analysis of γ-H2A.X in PK-15 cells treated with CdtABC or CdtAB^H161Q^C for 24 h, right shift of median fluorescence indicates a net increase of γ-H2A.X. (C) Quantitative Western blot analysis of γ-H2A.X in PK-15 cells treated with CdtABC or CdtAB^H161Q^C for 24 h. PAM cells (D) and PK-15 cells (E) were treated with CdtABC or CdtAB^H161Q^C for 36 h, and apoptotic and dead cells were stained with FITC-labeled annexin-V and PI, total cells were analyzed by flow cytometry.

Phosphorylation of H2A.X at serine 139 to γ-H2A.X is an early hallmark event after DNA double-strand breaks (DSBs) [22]. The role of γ-H2A.X is to recruit repair factors to the nucleus after DNA damage [23]. To further verify the DNase activity of CdtB in vivo, we analyzed the number of γ-H2A.X foci in CdtB-treated cells after 24 h. As shown in Fig 3A, the results showed that although the presence of CdtA and/or CdtC significantly increased the number of γ-H2A.X foci, CdtB alone was capable of generating γ-H2A.X foci in PK-15 cells. However, CdtA and CdtC did not activate the phosphorylation of H2A.X unless CdtB was present. The same trend was found with flow cytometry analysis in that there was an obvious increase in fluorescence intensity for γ-H2A.X after treatment with CdtB, CdtA/B, CdtB/C or CDT holotoxin (CdtA/B/C). Cells exposed to the CDT holotoxin had the strongest fluorescence (Fig 3B). Quantative Western blotting (Fig 3C) also found the same trend, treatment with CdtB alone resulted in increased expression of γ-H2A.X comparedto untreated control cells. This result is consistent with the quantitative analysis of flow cytometry (Fig 3D). Addition of CdtA and/or CdtC to CdtB treated cells resulted in greater expression of γ-H2A.X. In contrast, when the cells were exposed to mutant CdtB^H161Q^ together with CdtA and CdtC, no enhanced γ-H2A.X expression was found (Fig 4A, 4B and 4C). Collectively, these results show that CdtB has DNase activity in vitro and vivo and directly induces DSBs in PK-15 cells, and addition of CdtA and/or CdtC significantly enhanced the capability of CdtB to generate DSBs.

### CdtB-induced cell cycle arrest

**Fig 5:**
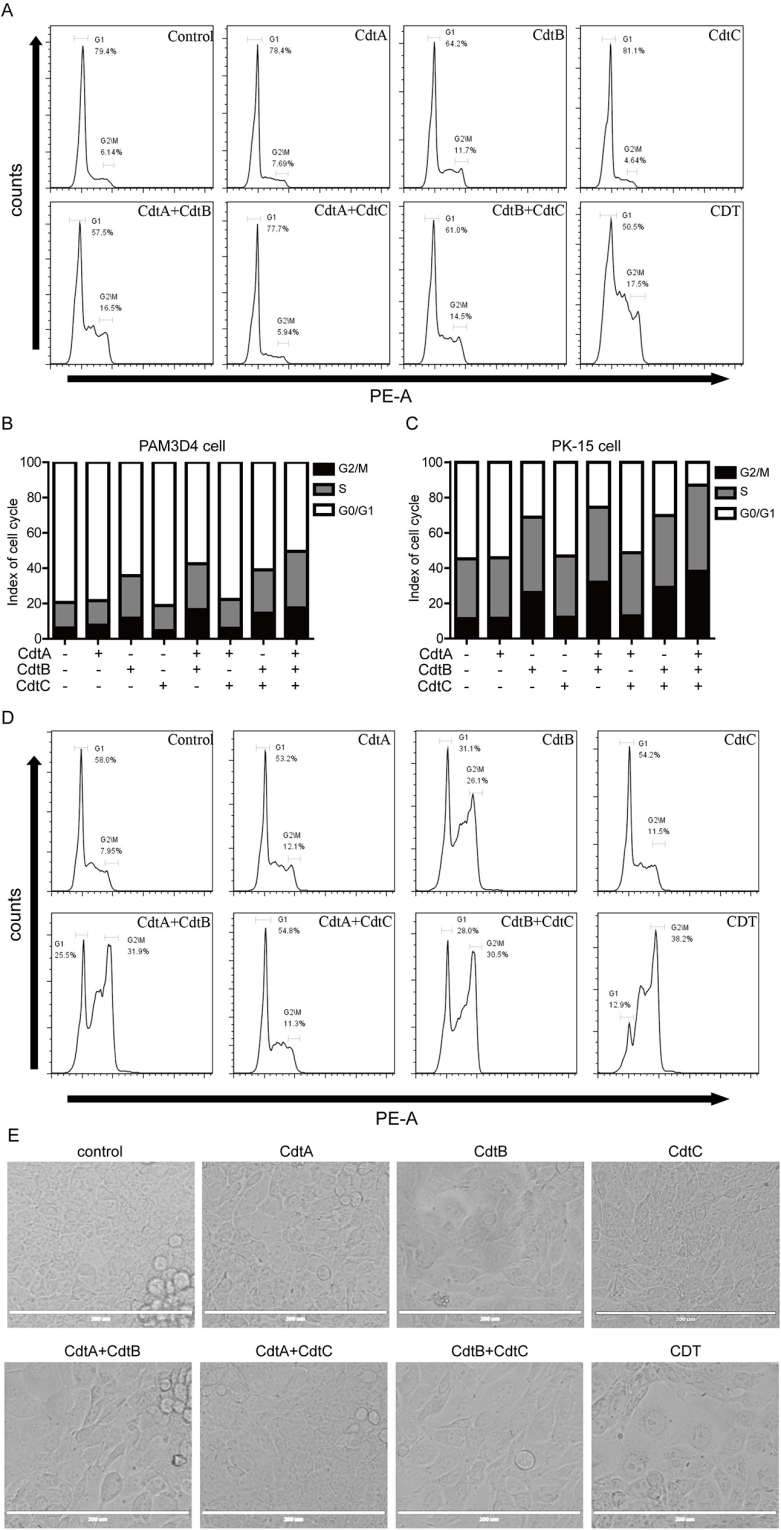
CdtB induced cell cycle arrest. PAM cells **(**A and B) and PK-15 cells (C and D) were treated with CDT for 24 h, and then DNA contents were analyzed by flow cytometry. (E) PK-15 cells were treated with CDT for 24 h, then cells were observed in a AMG EVOS F1 microscope (X 200 magnification), scale bar = 200 μm.

To detect whether CDT subunits induce cell cycle arrest, PAM and PK-15 cells were treated with different subunits of CDT for 24 h, collected and analyzed by flow cytometry. The results showed that when PAM cells were exposed to the CdtB, CdtA/B complex, CdtB/C complex or the CDT holotoxin, the percentage of cells in G2/M phase significantly increased: 11.7%, 16.5%, 14.5%, and 17.5%, respectively, compared to control cells 6.14% (Fig 5A and 5B).

When PK-15 cells were exposed to CdtB, CdtA/B complex, CdtB/C complex or the CDT holotoxin, a significant increase in the percentage of cells in G2/M phase was also found: 26.1%, 31.9%, 30.5%, and 38.2%, respectively. In contrast, the percentage of G2 control cells was 7.95% (Fig 5C and 5D). At the same time, to determine whether CDT could induce cell distention, PK-15 cells were treated with different subunits of CDT for 24 h. As shown in Fig 5E, cells treated with CdtA/B, CdtB/C or the CDT holotoxin became significantly larger than control or individual CDT subunits treated cells. These data suggest that CdtB alone is capable of inducing cell cycle arrest, and that CdtA and/or CdtC enhance the ability of CdtB (with CDT holotoxin exhibiting maximum activity) to induce G2 arrest and cell distention.

### CDT-induced cell apoptosis

**Fig 6:**
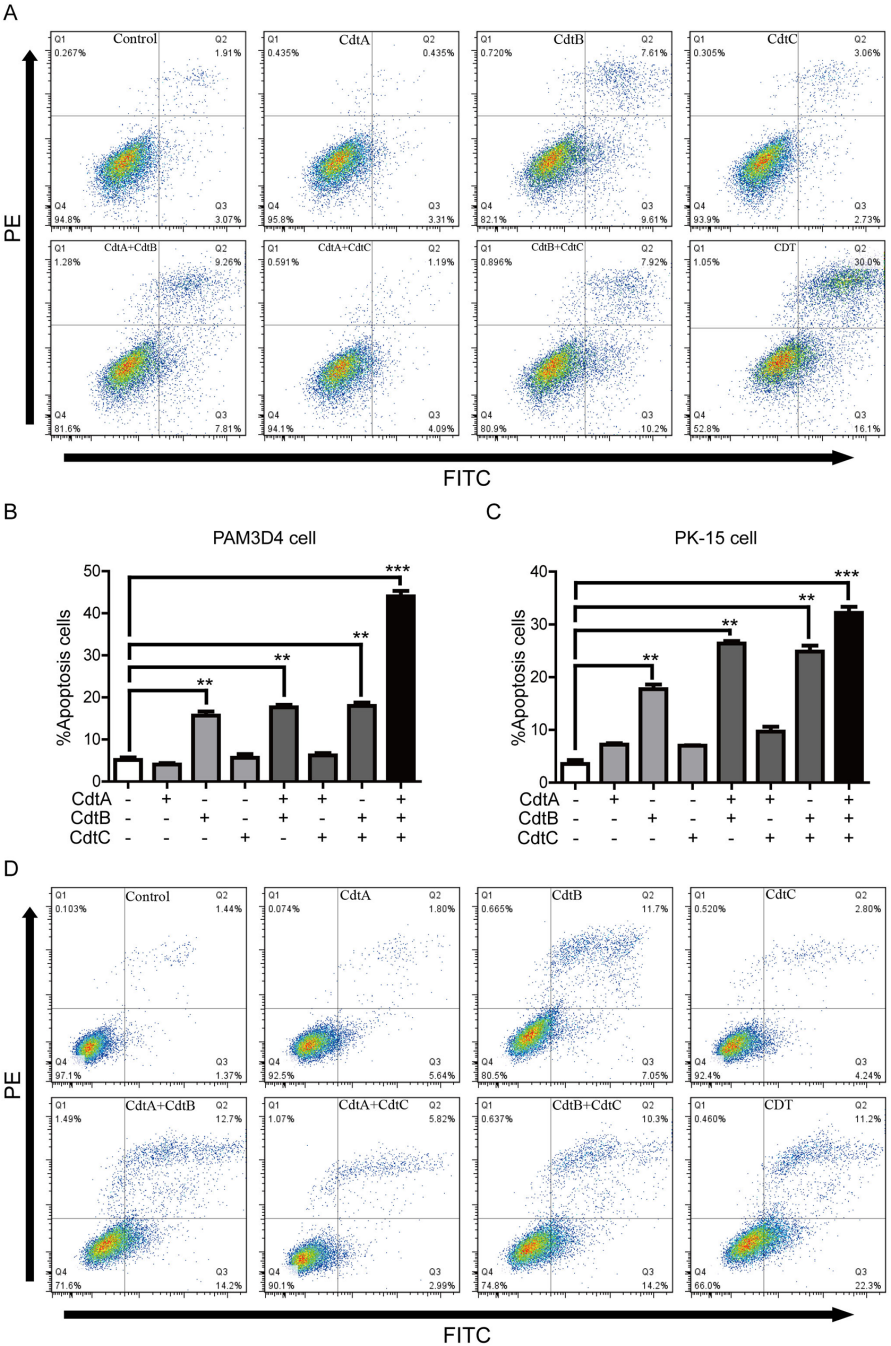
CdtB induced cell apoptosis. PAM cells (A and B) and PK-15 cells (C and D) were treated with CDT for 36 h, and apoptotic and dead cells stained with FITC-labeled annexin-V and PI, total cells were analyzed by flow cytometry.

Activation of apoptosis is a classical manner to eliminate damaged cells when DNA damage is irreversible. To detect whether CDT could induce host cell apoptosis, both PAM and PK-15 cells were exposed to those purified proteins and apoptosis monitored by flow cytometry at 36 h. The results showed that there was a significant increase in the level of apoptotic cells when PAM cells were exposed to CdtB (17.2%), CdtA/B (17.1%), CdtB/C(18.1%) and CDT holotoxin (46.1%), when compared to control cells (4.9%) (Fig 6A and 6B). Similar results were found with PK-15 cells (% apoptotic cells in brackets): CdtB (18.7%), CdtA/B (26.8%), CdtB/C (24.5%), and CDT holotoxin (33.5%). compared to2.8% for untreated cells (Fig 6C and 6D). PAM and PK-15 cells exposed to CdtAB^H161Q^C showed no increase in the percentage of apoptotic cells compared with untreated cells (Fig 4D and 4E). These data further demonstrate that CdtB alone is sufficient to induce apoptosis, and CdtA and/or CdtC enhance the genotoxicity of CdtB.

### CDT-induced cell apoptosis is p53-dependent

**Fig 7:**
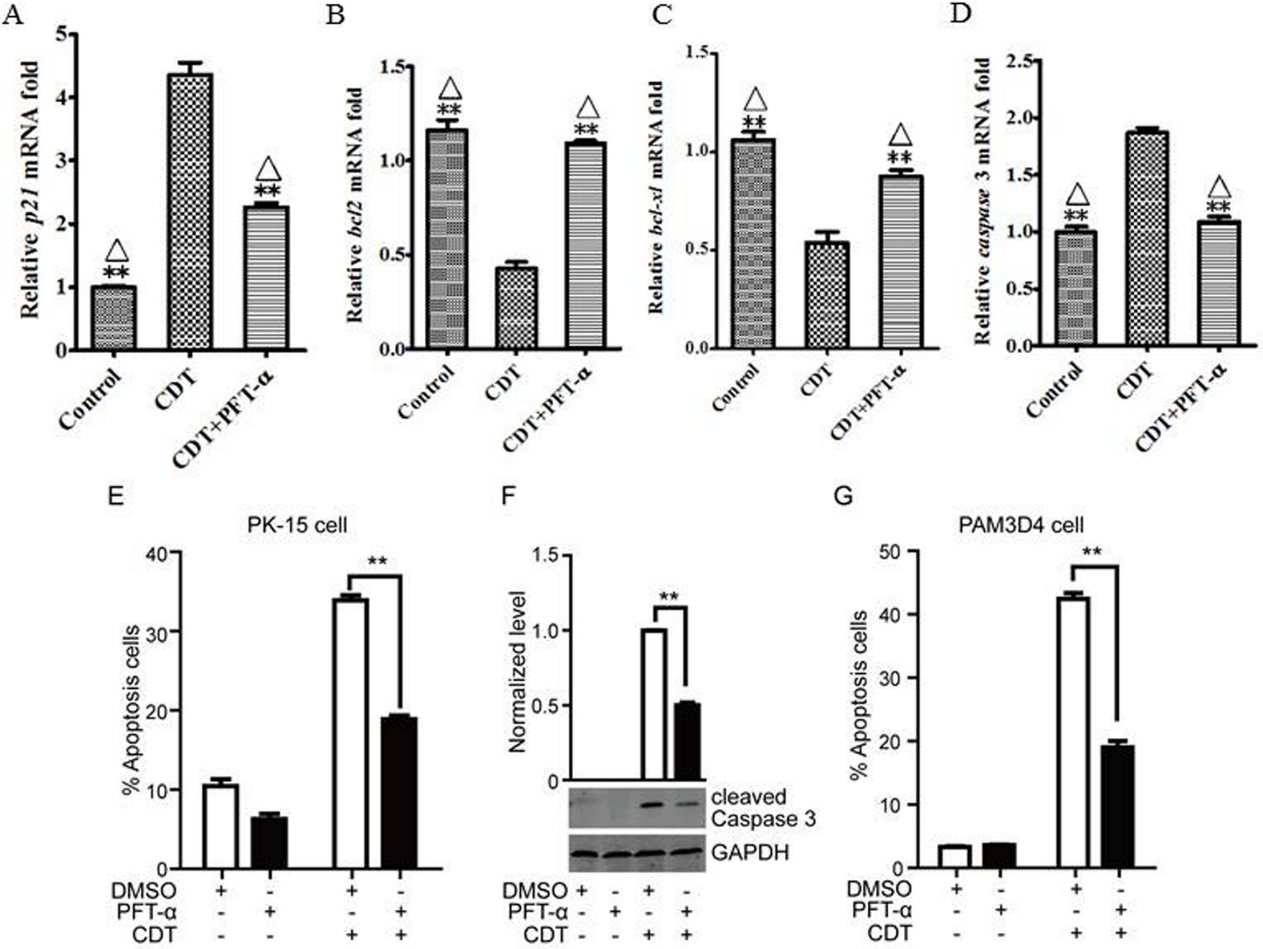
CDT-induced cell apoptosis is p53-dependent. (A, B, C, and D) PK-15 cells were treated with CDT for 36 h, total RNA extracted and the mRNA levels of *p21*, *Bcl2*, *Bcl-xl*, and *casp-3* were analyzed by real-time PCR. (E) PK-15 cells were treated with CDT and DMSO or co-incubated with CDT and PFT-α for 36 h, and apoptotic cells were stained with FITC-labeled annexin-V and PI, total cells were analyzed by flow cytometry. (F) PK-15 cells were treated with CDT and DMSO or co-incubated with CDT and PFT-α for 36 h, and the cleaved caspase-3 was detected by Western blot with anti-cleaved casp-3 pAb. Graphs show normalized level of cleaved casp-3, cells treated with DMSO and CDT were set as 1. (G) PAM cells were treated with CDT and DMSO or co-incubated with CDT and PFT-α for 36 h, and apoptotic cells stained with FITC-labeled annexin-V and PI, total cells were analyzed by flow cytometry.

The oncogene p53, a substrate of ATM/ATR (Ataxia Telangiectasia-mutated gene/ ATM and Rad3 related), is typically activated by the DNA damage response. To identify whether p53 was activated after cells were exposed to CDT holotoxin, the mRNA levels of *p21*, *Bcl2*, *Bcl-xl* and *casp-3* (which are regulated by p53) were analyzed at 36 h. The results showed the transcription level of *p21* (Fig 7A) and *casp-3* (Fig 7D) were 3.2 and 2 fold higher than the control respectively, the mRNA levels of *Bcl2* (Fig 7B) and *Bcl-xl* (Fig 7C) decreased to half of the control. However, when pifithrin-α (PFT-α), a specific inhibitor of p53, was added with CDT, the expression levels of these four genes were similar to that of the untreated control group. These data indirectly suggest p53 was activated by CDT holotoxin. In contrast, the apoptosis induced by CDT holotoxin was significantly attenuated by PFT-α as was expected, the percentage of apoptotic cells decreasing from 34.5% to 17% (PK-15 cells) and 43% to 19% (PAM cells) (Fig 7E and 7G). Furthermore, compared with CDT holotoxin treated cells, a great reduction of cleaved *casp*-3 was observed in cells exposed to both PFT-α and CDT holotoxin (Fig 7F). Taken together, these data show that CDT-induced cell apoptosis is p53 dependent.

## Discussion

*H*. *parasuis* has recently re-emerged as one of the major causes of nursey mortality in pigs. The mechanism of cell cycle arrest and apoptosis caused by HparCDT was still not been described clearly. In this study, we cloned and expressed the CdtA-C subunits of *H*. *parasuis* and determined the mechanism of their effects individually or combined on porcine cells. Predsi and SIGNAL-BLAST analyses predicted that the first 19, 21 and 19 aa’s at the N-terminal of CdtA, CdtB and CdtC, respectively are signal peptides. Subsequently, each subunit of the HparCDT was successfully expressed without the predicted signal peptides in soluble form. The N-terminal truncated CdtB had, as predicted for the wild-type, DNase activity.

All known CDT holotoxins have an AB_2_ structure where CdtB is the catalytic A unit and the binding B unit is composed of CdtA and CdtC [13]. It is thought that the CdtB subunit enters the cell with the help of CdtA and CdtC [6]. The tripartite model was confirmed despite the fact that CDT-specific phenotypes were reported in some cases in lack of A and / or C subunit. Taieb et al [24] using the same strategy, as in the present study, confirmed that in the case of *E*. *coli* CDT-V, as with other investigated CDT types, both CdtA and CdtC are necessary for the toxin to be fully functional [25]. Similarly the *A*.*actinomycetemcomitans* CDT was found to be functional, but with a reduced titre, when lacking the CdtA [12] or the CdtC subunit [26]. In harmony with the present study slight CDT activity was reported by *H*.*ducreyi* when lacking the CdtA subunit [27]. Similarly, *C*. *jejuni* CdtB caused the G2 arrest and cell distension when microinjected into the cytoplasm of target cells [28]. It is also important that that *Salmonella Typhi*, which does not express either CdtA or CdtC subunits, uses a different internalisation pathway to deliver the enzymatic CdtB subunit directly into the host cell [29]. The toxicity dependents on transcription of the *pltA* (pertussis-like toxin) and *pltB* genes whose products form with CdtB a tripartite complex known also as Typhoid toxin [30]. In this study, the CdtB from *H*. *parasuis* could induce the phosphorylation of H2A.X, G_2_ arrest and apoptosis in both PAM and PK-15 cells. Neither CdtA nor CdtC displayed the ability to inhibit cell cycle progression or induce cell apoptotic death independently in PAM cells although they induced a small amount of apoptosis in PK-15 cells. The ability of CdtB to induce G2 arrest, apoptosis and phosphorylation of H2A.X in PAM and PK-15 cells was enhanced in the presence of CdtA and CdtC. Exposure of PAM and PK-15 cells to both CdtA and CdtC lead to a significant increase in CdtB toxin activity.

Recent research has shown that virulent strains of *H*. *parasuis* co-localize with macrophages or neutrophils and can induce a delay in activation [31, 32]. These two studies demonstrate that CDT can induce proliferation detention and apoptotic death in both PK-15 and PAM cells. A previous study showed that the CdtB subunit was expressed by all the *H*. *parasuis* reference strains and 109 clinical isolates, and indicated that CDT is a conservative putative virulent factor of *H*. *parasuis* [16]. However, the molecular mechanism of *H*. *parasuis* CDT-induced cell cycle arrest and cell apoptosis has not been fully elucidated so far. Most reports are consistent with cell cycle arrest and apoptosis being directly induced by DSB [33–38]. However, few reports favor the opposite mechanism that cell cycle arrest and apoptosis lead to DNA fragmentation [39]. Our results showed that PK-15 and PAM cells treated with CdtB exhibited significant apoptotic death at 36 h, which is 12 h after CdtB-induced cell cycle arrest. In addition, CdtB has DNase activity in vitro and could induce the formation of γ-H2A.X foci when cells were exposed to CdtB for more than 12 h. All these data indicate that CdtB-induced DNA damage precedes cell cycle arrest and apoptosis in both PK-15 and PAM cells and is consistent with other studies [33–38] as discussed above.

Previous studies have shown that the cell apoptosis pathways induced by CDTs vary with bacterial strain and host cell type. For instance, the CDT from *H*.*ducreyi* induces cell cycle arrest and apoptosis in a p53-dependent way in ATM wild type cells, and changes to a p53-independent way in ATM-deficient cells [15]. The CDT of *H*. *hepaticus* induces cell apoptosis via the mitochondrial pathway[40]. In our study, using PFT-α to prevent the tumor suppressor protein p53, the percentage of apoptotic cells markedly decreased. Meanwhile, the mRNA levels of *Bcl-2* and *Bcl-xL* were significantly down-regulated, which is consistent with the finding that down regulation of Bcl-XL activity through deamidation is critical to cell apoptosis caused by DNA damage[41]. Therefore, we speculate that ATM/ATR kinases were phosphorylated and activated the tumor suppressor protein p53 which in turn mediated CDT-induced apoptosis by regulation of the proteins of Bcl-2 family and activation of caspase-3.

A previous study showed that *H*. *parasuis* induced newborn pig tracheal cells apoptosis via a caspase-3 dependent pathway, which was not due to lipooligosaccharide [42]. Overall, our results suggest that CDT is a major virulence factor of *H*. *parasuis* causing apoptosis. We speculate that it is the main virulence factor of *H*. *parasuis* causing apoptosis but this remains to be determined.

## Supporting information

S1 Fig The prediction of HparCDT signal peptides. (TIF)

S2 Fig Expression and purification of CdtB^H161Q^. (TIF)
