# Supplementary figures and images for: Haemophilus parasuis cytolethal distending toxin induces cell cycle arrest and p53-dependent apoptosis
Source: PLoS One. 2017 May 18;12(5):e0177199. doi: 10.1371/journal.pone.0177199 (PMC5436662; doi:10.1371/journal.pone.0177199)

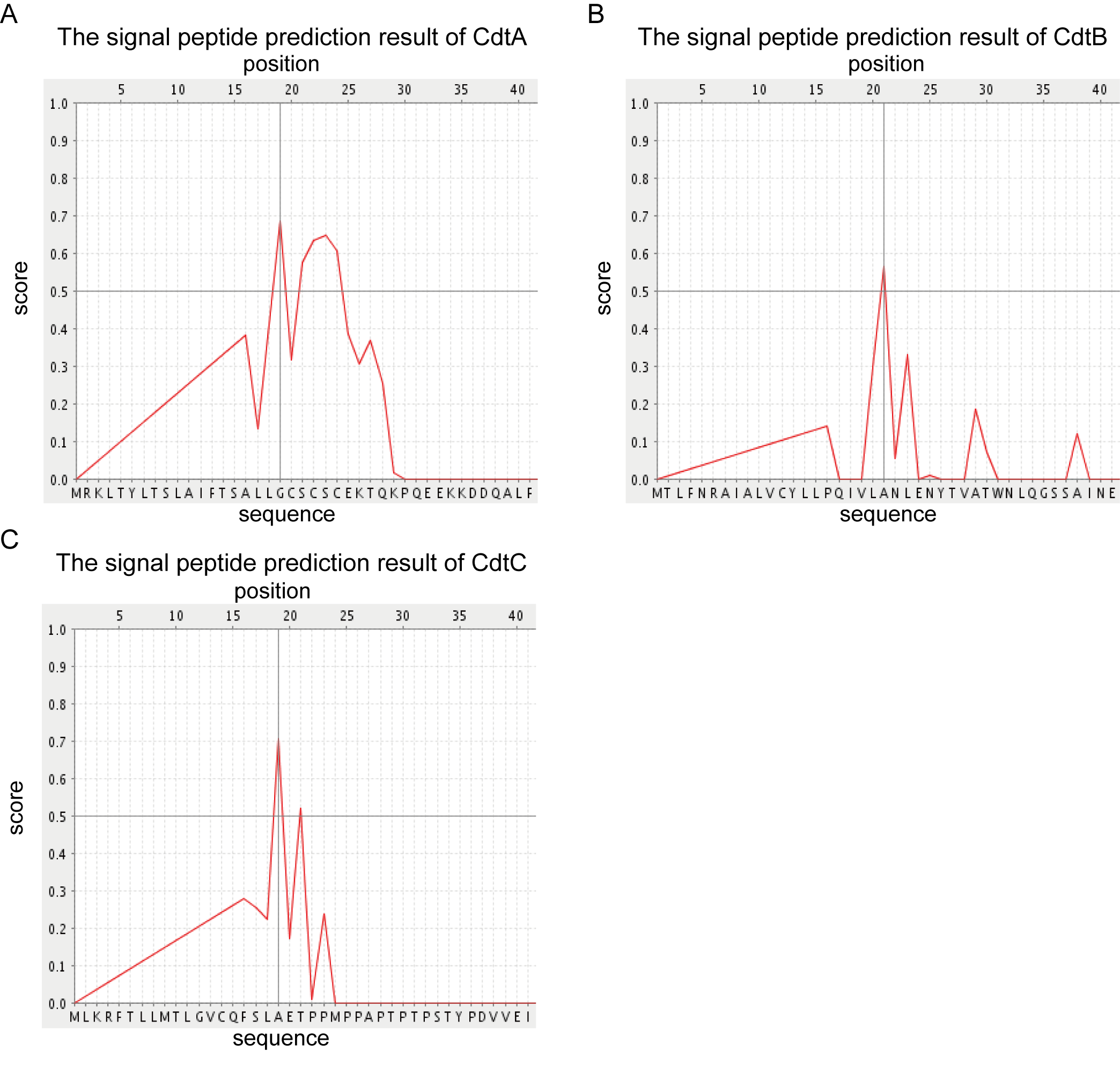

Supplement: S1 Fig — (TIF) [file pone.0177199.s001.tif]

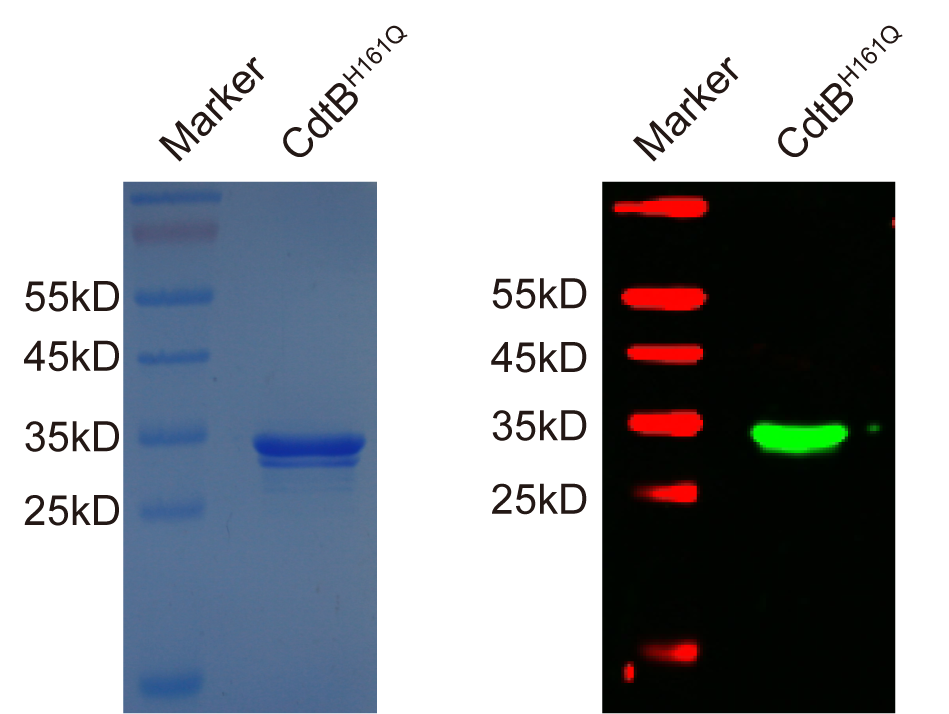

Supplement: S2 Fig — (TIF) [file pone.0177199.s002.tif]
